# Proteomic High Affinity Zn^2+^ Trafficking: Where Does Metallothionein Fit in?

**DOI:** 10.3390/ijms18061289

**Published:** 2017-06-17

**Authors:** David H. Petering, Afsana Mahim

**Affiliations:** Department of Chemistry and Biochemistry, University of Wisconsin–Milwaukee, Milwaukee, WI 53217, USA; aamahim@uwm.edu

**Keywords:** zinc, metallothionein, proteome, zinc trafficking, zinc protein, zinc signaling

## Abstract

The cellular constitution of Zn-proteins and Zn-dependent signaling depend on the capacity of Zn^2+^ to find specific binding sites in the face of a plethora of other high affinity ligands. The most prominent of these is metallothionein (MT). It serves as a storage site for Zn^2+^ under various conditions, and has chemical properties that support a dynamic role for MT in zinc trafficking. Consistent with these characteristics, changing the availability of zinc for cells and tissues causes rapid alteration of zinc bound to MT. Nevertheless, zinc trafficking occurs in metallothionein-null animals and cells, hypothetically making use of proteomic binding sites to mediate the intracellular movements of zinc. Like metallothionein, the proteome contains a large concentration of proteins that strongly coordinate zinc. In this environment, *free* Zn^2+^ may be of little significance. Instead, this review sets forth the basis for the hypothesis that components of the proteome and MT jointly provide the platform for zinc trafficking.

## 1. Overview of Zinc Trafficking

Zinc is a required nutrient for all forms of life. In mammalian organisms, as many as 3000 proteins may require Zn^2+^ to support their three-dimensional structures and/or biochemical functions [1,2]. Such proteins represent the endpoint of complex, only partially understood *trafficking* pathways that convey Zn^2+^ from external nutrient sources into organisms, then into cells through the agency of membrane transporters, and finally into contact with apo-Zn-proteins where metal ion binding occurs to constitute native Zn-proteins [3].

**Figure 1:**
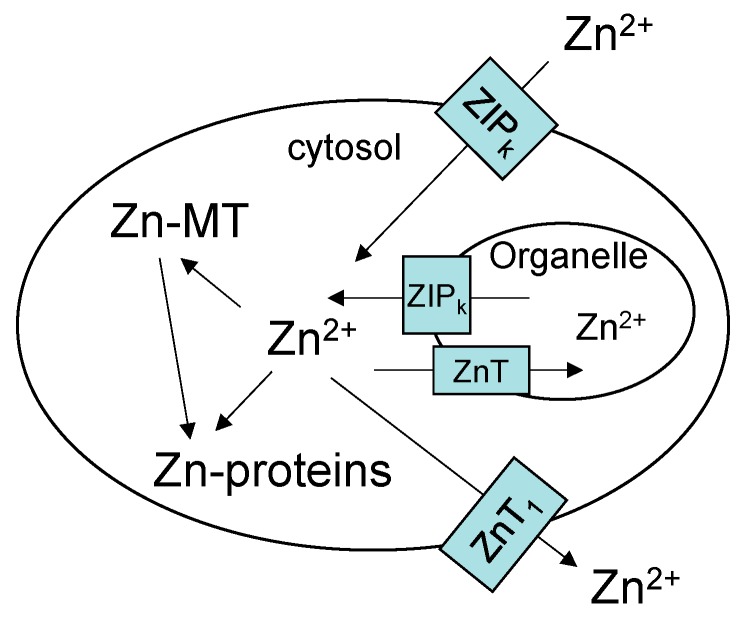
Contemporary view of cellular zinc trafficking [6,13,14]. Transporters move Zn^2+^ into cells and between compartments. Metallothionein (MT) controls the concentration of *free* Zn^2+^ that supplies zinc to proteins. Zn-MT may also contribute to trafficking of Zn^2+^ to Zn-proteins.

Studies over the past two decades have identified two families of Zn-transport proteins, ZIP and Zn-T, that escort Zn^2+^ into and out of mammalian cells and subcellular compartments [4,5,6] (Figure 1). Numerous reports document their key role in determining the intracellular availability of Zn^2+^ and the deleterious impact of mutations in Zn-transporter genes upon zinc-dependent processes [7]. Moreover, it is increasingly evident that the expression of at least some of these genes is under regulatory control, in part by the concentration of Zn^2+^ to which cells are exposed [8].

The trafficking of intracellular Zn^2+^ to its final sites of binding in Zn-proteins has remained relatively unexplored. Using the example of the constitution of Cu-proteins, it is hypothesized that upon transport into a cell, Zn^2+^ encounters metal-binding ligands (L_m_) and undergoes one or more ligand substitution reactions that culminate in the formation of functional Zn-proteins (Zn-P_n_) [9]:

(1) $$\mathrm{Zn}-{\mathrm{ZIP}}_{k}\overset{L_{1}}{\underset{\leftarrow }{\to }}n{\mathrm{Zn}-L}_{1}+{\mathrm{ZIP}}_{k}\overset{L_{2}}{\underset{\leftarrow }{\to }}n{\mathrm{Zn}-L}_{2}{+L}_{1}...\overset{L_{m}^{}}{\underset{\leftarrow }{\to }}n{\mathrm{Zn}-L}_{m-1}^{}{+L}_{m-1}\overset{P_{n}}{\underset{\leftarrow }{\to }}n{\mathrm{Zn}-P}_{n}{+L}_{m}$$

In the case of copper trafficking in yeast and mammalian cells, L_1_–L_m_ represent a limited number of specific chaperone proteins that conduct Cu from its plasma membrane transporter to a small set of Cu-proteins by associative ligand substitution processes. Clearly, individualized ligand substitution pathways for the formation of the large array of zinc proteins cannot be built with this same level of selectivity, as that would require as many as 3000 or more chaperone proteins targeting different zinc protein structures. This paper considers alternative hypotheses to explain the trafficking of Zn^2+^ based on past and current studies.

The difficulty of this task lies first of all in the complexity of intracellular Zn^2+^ metabolism that involves the delivery of zinc to thousands of metalloproteins. Second, in comparison with the understanding about the widespread use of Zn^2+^ as a co-factor in protein structure and function, there is a paucity of information about Zn-binding proteins that may serve as participants in Zn^2+^ trafficking [10]. Third, the analytical tools needed to track the cellular movement of Zn^2+^ remain underdeveloped [11]. Thus, portrayals of zinc trafficking acknowledge the important role of ZIP and ZnT transporters, but have little to add inside the cell beyond *free* zinc (Zn^2+^) and a protein that researchers have been preoccupied with for the past 60 years, metallothionein (MT) (Figure 1) [4,5,6,7,8,12,13,14].

MT pops into view in many different cells and tissues with its concentration or complement of Zn^2+^ changing significantly depending on the physiological or pathological context [15,16,17,18]. It may be a transient storage site for Zn^2+^ or a Zn^2+^ chaperone protein (Reaction (1)), but it cannot be the only structure that supports the flexible trafficking of Zn^2+^ to, from, and between the multitude of Zn-proteins.

Where does MT fit into cellular Zn^2+^ metabolism? The hypothesis will be developed that Zn^2+^ trafficking occurs through the general operation of Reaction (1). It involves the joint, interactive participation of high affinity Zn^2+^ binding sites provided by the proteome and metallothionein.

## 2. Chemical Properties of Metallothionein Related to Zn^2+^ Trafficking

The background for considering the role of Metallothionein I and II in the mammalian intracellular trafficking of zinc begins with an understanding of its chemical properties. MT is a small, sulfhydryl-rich protein containing 20 cysteinyl residues that can bind multiple metal ions, including nutritionally essential Zn^2+^, Cu^1+^, and a variety of non-physiological metal ions such as the toxic metal ions Cd^2+^, Hg^2+^, and metals with pharmaceutical properties, like Pt^2+^ and Bi^3+^ [19,20]. Because it coordinates conspicuous amounts of a variety of metal ions that cells encounter, attention has been focused on its metal coordination, metal exchange, and ligand substitution properties. In addition, in some circumstances the concentration of MT is large enough that its aggregate of thiol ligands is comparable in size to the proteomic and low molecular weight glutathione (GSH) pools of sulfhydryl groups [21]. In such instances, MT has the potential to contribute quantitatively to the cell’s redox chemistry [22].

MT that is saturated with divalent metal ions (M) segregates them between two domains (α and β), in which either a M_4_S_11_ or M_3_S_9_ metal-thiolate cluster, respectively, occupies the interior of one of the folded domains [19,23]. In early studies, the titration of apo-MT with Zn^2+^ in the presence of a non-specific protease resulted in stable products that were either Zn_4_S_11_-α or Zn_7_-MT [24].

(2) $$\mathrm{Apo}-\mathrm{MT}+n{\mathrm{Zn}}^{2+}\overset{\mathrm{protease}}{\to }{\mathrm{Zn}}_{n}-\mathrm{MT}\;(n\;=\;4\;\mathrm{or}\;7)$$

These findings, together with the knowledge of the structure of the clusters and their multiple sulfhydryl groups bridging between and connecting bound metal ions, supported the hypothesis that the protein structure is stabilized when one or both metal-thiolate clusters are formed.

The kinetics of formation of Zn_7_- and Cd_7_-MT have been characterized using stopped flow instrumentation [25]. Apo-MT and metal ions were mixed and allowed to react for increasing times before EDTA was added to quench the reaction. Three-sevenths of the overall reaction was too rapid to measure. The rest quickly formed an observable species that was thought to be M_4_S_11_-α, a structure slowly degraded by EDTA. Thus, in the presence of excess metal ions, stable domain structures fully occupied with metal ions are favored.

M_7_-MT undergoes facile ligand substitution- and sulfhydryl-based reactions. In a variety of reactions involving M_7_-MT, the kinetics are biphasic with each cluster thought to be responsible for one of the kinetic steps [26,27,28,29]. This implies that the rate-limiting reactions involve an attack on intact clusters. Once these structures are compromised, the remaining, bound metals or thiol groups react more rapidly.

This property has been used to examine the stability constants of Zn^2+^ in Zn_7_-MT [30,31]. Assigning each cluster to a kinetic step in a ligand substitution reaction and using the extent of reaction of each one to calculate equilibrium constants, the stability constant per Zn^2+^ at pH 7 in each cluster centered closely on 10^11.2^ M^−1^:

Zn_3_-β + 3L ⥂ 3Zn-L + β (faster phase) (3)

Zn_4_-α + 4L ⥂ 4Zn-L + α (faster phase) (4)

Recent analysis of the binding affinity of metals for MT has demonstrated that Zn*_n_*-MT (*n* ≠ 4 or 7) can exist not just protein with fully occupied clusters [32]. Accordingly, the titration of MT by Zn^2+^ is characterized by seven stability constants, which at pH 7 range from 10^11.8^ to 10^12.5^.

The magnitude of these equilibrium constants indicates that on a thermodynamic basis apo-MT should be able to compete for Zn^2+^ with at least some native Zn-proteins [10,33]. Conversely, apo-proteins with strong affinity for Zn^2+^ may be able to sequester it from Zn*_n_*-MT (*n* = 1–7), as in Reaction (1). For example, multiple experiments have revealed that apo-carbonic anhydrase (stability constant at pH 7 = 10^11.4^) successfully competes for Zn^2+^ bound to Zn_7_-MT [26,32,34,35,36]. The reconstitution of the zinc-finger protein, Zn-tramtrack from apo-tramtrak and Zn_7_-MT has also been shown [37].

The findings and conclusions above have been challenged by experiments showing that Zn_7_-MT can restore Zn^2+^ and activity to several proteins that display only moderate binding strength with Zn^2+^ [35,38,39]. In some of these cases, MT donated only one zinc to the acceptor protein [38,39]. Later, it was found that the protein preparation used in these studies contained one Zn^2+^ characterized by a stability constant at pH 7 of 10^7.8^, as well as six others with stability constants centered on 10^11^ [40]. Apparently, protein activation by Zn^2+^ related to this low affinity site. Recently, it was discovered that a step in the preparation of the protein involving its incubation at pH 2 converted native MT with seven strong binding sites to this modified form [31]. Thus far, there is no evidence that cellular conditions can produce this altered species. Instead, MT apparently serves as a high affinity ligand for all of its bound Zn^2+^ ions.

The behavior of apo-MT in its titration with Co^2+^ contrasts with Reaction (2) [25,41]. Acting as a surrogate for Zn^2+^, the reaction of Co^2+^ with apo-MT was monitored first by electron paramagnetic resonance spectroscopy and later by the differential reactivity of metal-free and bound sulfhydryl groups. It occurs in step-wise fashion, not in a concerted all-or-nothing process that generates only fully formed clusters:

Co^2+^ + apo-MT ⥂ Co-MT ⥂ Co_2_-MT ⥂ Co_3_-MT ⥂ Co_4_,S_11_-MT… (5)

An early titration of apo-MT with Zn^2+^ monitored with electrospray mass spectrometry (ESI) suggested that cluster unsaturated species of Zn*_n_*-MT (*n* = 1–3, 5, 6) form along with clusters fully occupied with Zn^2+^ [42]. Subsequently, an elegant series of experiments by Stillman and his students, also using ESI, strongly supported the stepwise formation of Zn_7_-MT from Zn^2+^ and apo-MT [32,43,44]. In particular, when apo-MT, apo-CA, and various concentrations of Zn^2+^ were mixed, the metal ion distributed itself among Zn-CA and Zn*_n_*-MT (*n* = 1–7) such that equilibrium constants for the binding of each Zn^2+^ to MT could be determined as described above [32]. They ranged between 10^12.5^ to 10^11.8^ for the first and seventh zinc ion, respectively.

The large thermodynamic stability of Zn_7_-MT supports its capacity to bind extra Zn^2+^ that enters cells and suggests that it may compete for intracellular Zn^2+^ (Figure 1). In contrast, its unusual kinetic reactivity and demonstrated reaction with apo-zinc proteins raises the possibility that Zn*_n_*-MT might participate in zinc trafficking as in Reaction (1) [45]. How these properties reveal themselves in the cell is considered below.

## 3. Cellular Zn^2+^ and Its Milieu

Typical mammalian cells contain concentrations of zinc in the range of 100–500 µM [46,47]. Virtually all of it is thought to represent the collection of native Zn-proteins that populate the cell. Conditional stability constants for a few of them have been measured at pH 7 [10,33]. They range between 10^9^ and 10^12^ M^−1^. As discussed below, the pressure of zinc deficiency exerts little if any measurable effect on the concentration of Zn^2+^ in a number of tissues and cells, exclusive of that bound to metallothionein.

**Figure 2:**
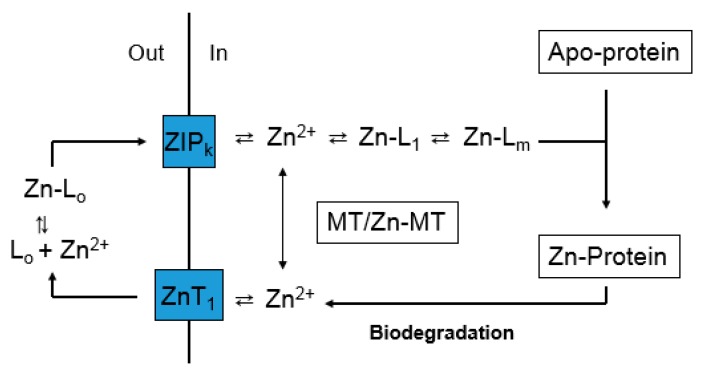
Hypothetical Zn^2+^ trafficking pathway based on Figure 1.

The large concentration of Zn^2+^ bound to Zn-proteins is mobile on some time scale because in the dynamics of synthesis and degradation of intracellular proteins, Zn^2+^ must be continually delivered to newly synthesized apo-Zn-proteins as it is released from proteins undergoing hydrolytic break-down (Figure 2). Potentially, this is a cyclic process with respect to Zn^2+^ and zinc binding ligands L_1-m_ (Reaction (1)), but may also involve cellular uptake and release of Zn^2+^ via transporters. In this diagram, it is assumed that L_1-m_ represent proteins or possibly small metal coordinating ligands such as glutathione (GSH). A role for MT is not specified; but it has been suggested that MT serves as an intermediate in the trafficking of zinc to apo-zinc proteins [26,45].

The functional groups of a number of amino acid side chains can serve as ligands for metal ions (amine, carboxyl, imidazole, thiol) [48]. It is not surprising, therefore, that proteins generally display affinity for a variety of metal ions. Maret and Krezel used the term *zinc buffering* to describe non-specific binding of Zn^2+^ by the cell’s complement of proteins and small molecules. To reveal this property, cytosol from human colon cancer (HT-29) cells was titrated with Zn^2+^ in the presence of a colorimetric sensor, Zincon (ZI), which has a modest stability constant for Zn^2+^ at pH 7 of 10^4.9^ M^−1^ [46]. On a cellular basis, 30 µM Zn^2+^ or about 10% of the size of the Zn-proteome reacted with the proteome before Zn-ZI began to form.

A similar experiment employed proteome from the sonicated supernatant of pig kidney LLC-PK_1_ cells [49]. In the presence of the fluorescent sensor, Newport Green (NPG) (*K*_Zn-NPG_ = 10^5^ M^−1^, pH 7), titration of the proteome with Zn^2+^ revealed a much larger complement of high affinity coordination sites that bind Zn^2+^ exclusively in the presence of NPG and an even greater concentration of low affinity sites with which NPG directly competes (Reactions (6) and (7)).

Proteome_high affinity_•Zn + S (ZI, NPG) ⥂ no reaction (6)

Proteome_lower affinity_•Zn + S ⥂ Zn-S + Proteome_low affinity_ (7)

Because these cells do not contain measurable metallothionein, it was evident that proteins other than MT contribute to the binding of Zn^2+^.

Titration of LLC-PK_1_ proteome with Zn^2+^ in the presence of another fluorescent probe, Fluozin-3 (FZ-3), which has a higher conditional stability constant for Zn^2+^ of 10^8.1^ M^−1^ at pH 7, resulted in the same general picture, except that FZ-3 competes with Proteome_high affinity_ for Zn^2+^ (Karim and Petering, unpublished information). Because of this, a conditional stability constant of Proteome_high affinity_ for Zn^2+^ could be estimated at 10^10^ M^−1^. The LLC-PK_1_ cell proteome contains a multitude of such binding sites for Zn^2+^, about 1.5 times more than are present in native Zn-proteins. Repetition of the experiments with ZI, NPG, and FZ-3 after treating the proteome with the thiol reactive agent N-ethylmaleimide quantitatively abolished high affinity sites, leaving only unperturbed, low affinity binding of Zn^2+^ by the proteome. Dithiodipyridine and nitric oxide also react with sulhydryl groups and have a similar qualitative impact on adventitious zinc binding by the proteome [50,51]. Apparently, strong binding sites for Zn^2+^ involve thiolate ligands. Not known, however, are the numbers, identities, and concentrations of the proteins that interact with Zn^2+^.

The Zn^2+^ binding strength and large size of the collection of proteins in the proteome_high affinity_ make clear that glutathione (stability constant for Zn^2+^ at pH 7 of 10^4.2^) does not play an independent role as an intermediate in Zn^2+^ trafficking (Reaction (1)) [52]. Instead, the model presented in Figure 2 portrays trafficking proteins (L_1-m_). Consistent with this emphasis, Zn^2+^ added to cells or cell lysates or mobilized from native Zn-proteins by displacement with Cd^2+^ associates with the proteome fraction not with glutathione or other low molecular weight molecules [53]. It is within this context that a role for metallothionein in Zn^2+^ trafficking must be considered and begins to make sense.

The findings above bring into question the significance of *free* zinc (Zn^2+^). The pool of Zn^2+^ in Figure 1 and Figure 2 has been invoked as the direct source of zinc for the synthesis of Zn-proteins, a participant in zinc signaling processes (see below), and as the agent of zinc toxicity [40,54]. Thus, the measurement of its concentration under various conditions using fluorescent zinc sensors (S) has received substantial attention [55,56]. Sensors are thought to react with *free* or *available* Zn^2+^ according to Reactions (8)–(10):

Zn^2+^ + S ⥂ Zn-S   (K) (8)

Zn-L + S ⥂ Zn-S + L    (K′)(9)

Zn-Protein + S ⥂ Zn-S + Protein   (K′′)(10)

in which L represents non-specific binding sites and Zn-Proteins are specific sites. Depending on the conditional stability constant of Zn-S (K), and the kinetics of its reaction with zinc-ligand complexes, S may be able to sequester Zn^2+^ from Zn-L or Zn-Protein sites as well as react with Zn^2+^. Thus, whether measurements with zinc fluorescent probes reflect the presence of Zn^2+^ or the accessibility of other pools of Zn^2+^ remains in doubt. Nevertheless, in unperturbed cells, the most that fluorescent probes detect is low nM to low pM zinc [57,58,59]. At such miniscule concentrations, and in comparison with the total Zn^2+^ in cells, it seems unlikely, if not impossible on a kinetic basis, that steady state concentrations of *free* Zn^2+^ can supply the metal ion needed to constitute new Zn-proteins in proliferative cells [3]. Therefore, Reaction (1) remains as the viable, hypothetical pathway that leads to the formation of Zn-proteins. More generally, the focus on *free* Zn^2+^ seems misplaced. In the equilibrium or steady state reactions that connect *free* and *bound Zn*^2+^, it is the structures with which *mobile* Zn^2+^ interacts that give rise to functional outcomes, and not *free*, unbound Zn^2+^.

## 4. Metallothionein and Zinc Trafficking—The Biological Context

Metallothionein was discovered as a cadmium-binding protein in horse kidney [60]. Within a decade it was clear that the protein was rapidly inducible and that copious amounts of hepatic Zn-MT could be accumulated upon elevated dietary intake of Zn^2+^, and lost rapidly upon imposition of zinc deficiency [61]. Soon, it was realized that high concentrations of metallothionein were present under normal physiological conditions. For example, fetal and neonatal bovine and human liver contains large amounts of Zn,Cu-MT, thought to serve as a mobilizable store of these metals for the developing organism [62,63]. Likewise, juvenile and adult rat kidney contains substantial pools of Zn,Cu-MT, for reasons that remain unclear [15]. Moreover, rats exposed to a variety of stresses, such as cold, pathogens, burns, chemicals, etc., quickly accumulate Zn^2+^ from plasma in hepatic MT as part of the general organismic stress response [16]. Such results focused attention on MT’s participation in Zn^2+^ trafficking as a dynamic storage location that might chemically exchange metal ions with other sites.

The constitutive presence or induction of MT protein also potentially introduces a large pool of sulfhydryl groups into the cell alongside those of glutathione and the proteome. As a consequence, MT may play an important role in the cell’s response to stress imposed by electrophilic reagents, such as oxidants. Numerous chemical and cellular studies document the reactivity of thiolate groups in Zn- and apo-MT with agents such as H_2_O_2_ vs. NO [21,22,64,65]. As anticipated, Zn^2+^ retards the reaction of sulfhydryl groups with electrophiles [25,64]. Conversely, oxidative modification of MT’s sulfhydryl groups renders bound Zn^2+^ available for trafficking reactions [66]. From this point of view, the involvement of MT in Zn^2+^ trafficking and in cellular redox chemistry become intertwined.

## 5. Zinc Trafficking in Metallothionein-Null Organisms and Cells

Inquiries into the functions and importance of metallothionein must recognize that gene knock-outs of MT I and II do not prevent the birth and survival of MT-null mice [67]. Clearly, MT is not required for zinc trafficking; cells can manage zinc metabolism without it. In this context, investigations with MT-null animals have revealed the protein’s significance in protecting cells faced with a host of stress conditions, including exposure to heavy metals such as Cd^2+^ and methylmercury, challenge by toxic oxidants and chemotherapeutic drugs with electrophilic properties, [68,69,70,71].

Laboratory animals live in hygienic conditions and consume super-optimal diets, commonly receiving, for instance, multiple times their daily requirement of Zn^2+^. Reproduction and survival of MT-null mice occurs under such conditions. In contrast, when pregnant MT-null mice were subjected to mild zinc deficiency that caused little harm to control fetuses, about 30% died or showed frank malformations [72]. Moreover, MT-null mice experienced reduced body weight gain and bone structure development with the effects intensifying as dietary zinc declined from normal to modestly deficient [73]. Evidently, the absence of MT compromised the handling of zinc within the mother and fetus and then in the growing offspring. These results signal a need to examine how MT plays a significant, but subtle, role in zinc trafficking.

Other reports also document the deleterious impact of the absence of MT I and II on the physiology of mice. The MT null phenotype alters neurological function and causes alterations in energy metabolism leading to modest obesity [74,75]. Transient down-regulation of MT inhibits cell cycle progression in proliferative breast cancer cells and causes apoptosis of ovarian and prostate tumor cell lines [76,77,78].

All of these results infer that the MT protein has important roles to play in a variety of cellular activities, a number of which are associated with zinc. However, a general short-coming of these reports is that the observations of physiological derangements in MT-null mice stand alone. Parallel studies have not been undertaken to understand the underlying chemistry of MT and zinc in control cells and their MT-null counterparts. As a result, mechanisms by which MT exerts its effects remain speculative.

## 6. Metallothionein, Cell Proliferation, and Zinc Deficiency

Normal fetal and neonatal/juvenile growth and development are highly reliant on the presence of an adequate supply of nutrient Zn^2+^ [79]. For example, in juvenile rats, plasma zinc concentration plummets within 24 h upon imposition of a zinc deficient diet [16]. Increase in body weight terminates in this same time frame. Kidney, which contains a readily measurable concentration of Zn,Cu-MT, also loses its complement of MT-bound Zn^2+^ during this period. In contrast, upon imposition of zinc deficiency for at least 30 days, the concentration of proteomic zinc other than what is bound to MT does not change in kidney or other tissues, such as liver [16]. After restoration of zinc to the diet, plasma and MT regain Zn^2+^ with the same kinetics, and the rats begin growing again. Thus, at this gross level of analysis, MT appears unique in its responsiveness to nutrient zinc status. Such observations sharpen the focus on metallothionein in relation to Zn^2+^ trafficking.

Cancer tumor growth is also keenly sensitive to dietary zinc status [80]. In an Ehrlich ascites carcinoma tumor model, cancer cells injected into the mouse peritoneum stimulate ascites fluid production that provides the cells with their nutrition [81,82]. The concentration of zinc in the fluid paralleled levels of dietary zinc; at low values, cell proliferation was inhibited. In contrast, over the range of normal to deficient concentrations of dietary zinc, the amount intracellular zinc did not measurably vary.

The involvement of metallothionein in supporting cell proliferation has also been established in a series of MT-knockdown and knockout studies [76,77,83,84]. With this association between MT, zinc, and cell proliferation established, studies have inquired into the mechanistic role of metallothionein in supporting zinc-contingent growth of organisms and cell populations.

Ehrlich ascites cells contain a steady-state pool of Zn-MT that accounts for about 15% of the cellular Zn^2+^ [85]. When placed in a zinc-deficient medium in vitro, MT lost Zn^2+^ rapidly with a first order half-time of 1 h, whereas the rest of the proteomic zinc declined slowly with an estimated *t*_1/2_ of 43 h [86]. The half-times for MT and proteome protein degradation were measured as ca. 5 and 11 h, respectively. The results demonstrated that the shift of Zn^2+^ out of MT was not rate limited by its biodegradation. In addition, the rate of loss of proteomic zinc was much slower than the turnover rate of the proteome’s complement of proteins. Not determined in this work was the fate of the zinc that was transferred out of MT. This experiment also revealed that proteomic Zn^2+^ was maintained despite the turnover of protein. Importantly, it supported the hypothesis that Zn-MT participates in cellular Zn^2+^ trafficking and that it does so through a chemical mechanism not passive protein turnover.

The rate limiting chemical reaction might involve dissociation of Zn^2+^ from MT or ligand substitution with competing binding sites for Zn^2+^:

Zn-MT ⥂ MT + Zn^2+^ (11)

Zn-MT + L_m_ ⥂ MT + Zn-L_m_ (12)

Because of the large conditional stability constants for the coordination of Zn^2+^ by MT, the kinetics of dissociation of Zn^2+^ from MT (Reaction (11)) must be very slow and cannot account for the swift depletion of MT-bound Zn^2+^ in this experiment [31,32]. Thus, Reaction (12) remains, and is well supported by chemical studies, showing the ligand substitution reactivity of MT [26,28,45]. This experiment distinguished the behavior of Zn^2+^ bound to MT from that associated with the proteome. The former behaved like a trafficking intermediate or labile store of Zn^2+^, and the latter as its steady state destination.

In vivo experiments examined the behavior of Ehrlich ascites cells in mouse peritoneum after transition of animals to a zinc-deficient diet. Ordinarily, the Ehrlich ascites tumor is lethal to mice about 17 days after injection of cells. In contrast, under zinc-limiting conditions, there was no gross evidence of tumor after 24 days, consistent with previous observations that zinc deficiency inhibits both normal tissue and tumor growth [81]. Dormant tumor cells contained 80% of the control level of proteomic zinc, essentially missing Zn^2+^ associated with MT, in agreement with the view that MT-bound Zn^2+^ is particularly labile in the face of changing external concentrations of zinc [87].

**Figure 3:**
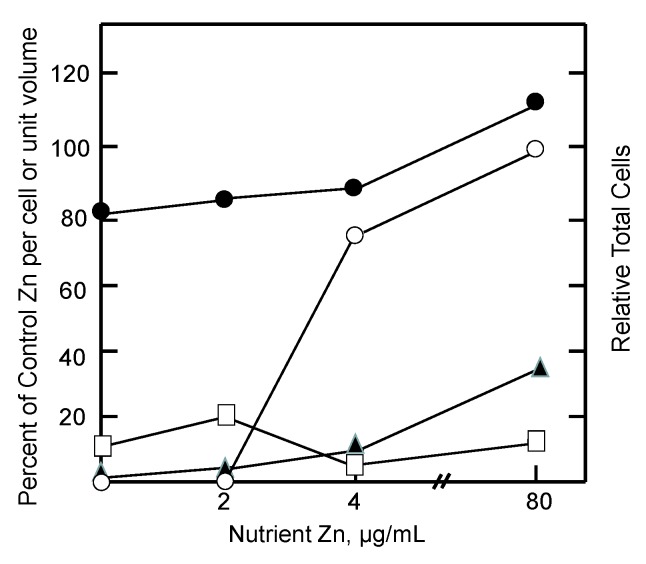
Response of tumor and host pools of zinc to graded restoration of nutrient Zn (adapted from [87]). Mice were held on a Zn-deficient diet for 2 weeks prior to injection of tumor cells. Measurements were made 6–9 days after reintroduction of nutrient Zn. Relative total cells (○) and relative proteomic Zn (●), MT Zn (▲), and ascites fluid Zn (□) per cell or unit volume.

During the reactivation of tumor growth by zinc, the extent of reentry of zinc into (i) ascites fluid, the immediate source of Zn^2+^ for the cells; (ii) the growing cell population; and (iii) intracellular proteomic and MT pools were assessed. As illustrated in Figure 3, measurements during days 6–9 after addition of zinc to the diet showed that tumor growth was restored by increasing concentrations of nutrient zinc. Evidently, the tumor acquired the requisite amount of zinc to support cell division. But the availability of zinc did not drastically alter the per cell proteomic zinc concentration. In contrast, even at the super optimal level of 80 µg/mL of dietary zinc, ascites fluid zinc remained depressed while zinc associated with MT was only partially restored to control levels. It was clear that if cells are to divide, they must acquire their total, functional complement of proteomic zinc. That was not the case for ascites fluid and MT. Instead, as in the in vitro cellular experiments described above, metallothionein behaved like an intermediate along the pathway of zinc from diet to the proteome as did ascites fluid. As such, the results are consistent with the zinc trafficking sequence,

Diet → Ascites fluid → MT → Proteome(13)

The behavior of metallothionein during the stress of zinc deficiency has been the subject of other studies, as well. O’Halloran and others discovered that MT mRNA increased markedly in fibroblast cells (LZA-LTK-) and animals upon their transfer into a zinc-deficient growth medium, suggesting a role for the protein in the response to the stress of limiting available Zn^2+^ to support proliferation [73,88]. These studies documented the inhibition of cell proliferation by the zinc deficit, but were unable to show that the MT protein concentration was correspondingly elevated.

Recent experiments addressed this question with three cell lines, LLC-PK_1_ (pig kidney proximal tubule), TE-671 (human rhabdomyosarcoma), and U-87 (human glioma) (Rana and Petering, unpublished information). The first two contain little MT. The third displays a significant, constitutive concentration of zinc-unsaturated MT. When each was shifted into a growth medium that was depleted of Zn^2+^, elevated concentrations of apo-MT appeared within 24 h that ranged between 1.6 and 4 times control levels. Coincidently, the proliferation of each cell line was halted without net loss of zinc from the cells.

**Figure 4:**
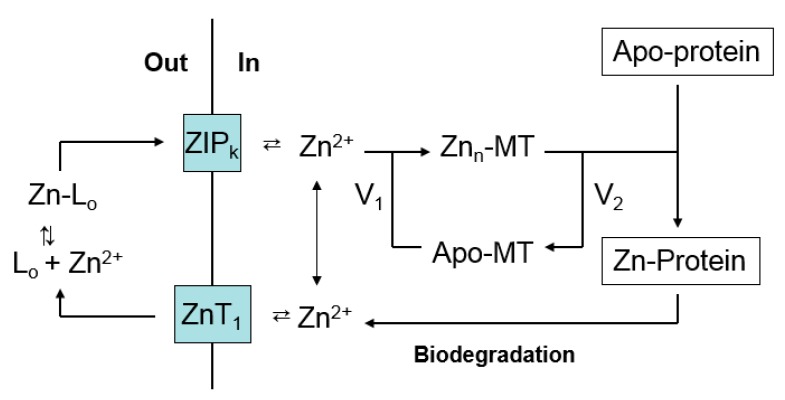
Cellular Zn^2+^ trafficking involving metallothionein. Based on Figure 3, the pathway emphasizes Zn-MT, but does not exclude other Proteomic intermediates (L_1-m_). *V*_1_ and *V*_2_ represent, respectively, the rates of formation of Zn-MT from apo- or undersaturated MT and the donation of Zn^2+^ to apo-proteins by Zn-MT.

These results are rationalized in relation to Figure 4. In the absence of nutrient zinc, the plasma membrane exporter of Zn^2+^, ZnT1, is down-regulated to prevent the loss of intracellular Zn^2+^, which becomes transiently mobile during the biodegradation of Zn-proteins [89]. In addition, in this model, the pool of intracellular zinc is sustained by up-regulation of MT concentration which both sequesters Zn^2+^ released by Zn-protein degradation and potentially donates it back to apo-Zn-proteins.

## 7. Metal-Unsaturated Metallothionein and the Trafficking of Zinc

The 3-dimensional structures of Cd_5_,Zn_2_-MT and Cd_7_-MT are determined by the interior metal-thiolate clusters, which constrain the folding of the polypeptide chain around them [19,90]. The conventional view has held that in the absence of metals, MT is unstructured and should be swiftly degraded in cells. Moreover, its destruction is necessary to prevent deleterious competition for Zn^2+^ between metal-unsaturated or apo-MT and native Zn-proteins. Nevertheless, it was discovered that numerous proliferative cell types contain readily measurable and sometimes large concentrations of constitutive apo- or unsaturated Zn-MT under normal growth conditions [91]. Subsequently, this finding was extended as well to non-proliferative tissues [92]. Moreover, a variety of agents that induce MT synthesis such as the stress hormone dexamethasone result in metal-unsaturated MT, as do conditions that cause the loss of metal ions from MT [93]. Detection of unsaturated Zn-MT is fully consistent with recent chemical studies showing that MT accommodates 1–7 Zn^2+^ ions [32,43].

Apo-MT and partially zinc-saturated protein display a similar overall shape to M_7_-MT, migrating during gel filtration chromatography like an ellipsoid (10 kDa) rather than a globular (6 kDa) protein [91,94]. That the apo-protein adopts a secondary structure is supported by experiments showing that the reactivity of cysteinyl sulfhydryl groups differs in native and denatured apo-MT, and that the rate of binding of Cd^2+^ to the protein also varies between these two states [95,96].

Considering the large stability constants that characterize the interaction of Zn^2+^ with MT (10^11–13^), it is important to understand how cellular apo-MT can co-exist with the extant Zn-proteome, and with the zinc-trafficking mechanism that provides zinc for Zn-proteins being synthesized during cell proliferation [31,32]. Remarkably, on a quantitative basis, apo-MT competes poorly, if at all, with isolated Zn-proteome from LLC-PK_1_ cells, even though a number of organic chelating agents with conditional stability constants ranging from 10^15^ to 10^9^ at pH 7 are able to extract up to 30% of proteomic zinc under similar conditions (Reaction (14)) [97].

Zn-Proteome + L ⥂ Zn-L + Proteome(14)

Evidently, apo-MT is kinetically inert to ligand substitution in reactions of this sort. Possibly, intermediate L-Zn-protein adducts are crucial for the mechanism of substitution. Whereas smaller multidentate ligands make such intermediates achievable, the steric bulkiness of apo-MT inhibits this stage of the reaction. In support of this view, it has been demonstrated that the partial MT sequence 51–60, including four cysteinyl thiolates that normally bind to a single Zn^2+^ in Zn_7_-MT, sequesters Cd^2+^ from Cd-carbonic anhydrase much more rapidly than the more sterically hindered apo-MT [98].

Apo-MT does react with and inactivate the zinc-finger protein, Zn_3_-Sp1, which utilizes three tandem cys_23_his_2_ Zn-fingers to bind to its cognate DNA [97].

Zn_3_-Sp1 + apo-MT **⥂** Zn_3_-MT + apo-Sp1 (15)

Presumably, the relatively accessible zinc coordination sites of Zn_3_-Sp1 favor ligand substitution, as discussed above. Comparison of this reaction in the presence and absence of a GC-rich DNA binding sequence for human Zn_3_-Sp1 revealed that DNA protected the protein against loss of Zn^2+^ to MT [97]. This observation is consistent with the finding that specific DNA binding also protects Zn_3_-Sp1 against reaction with powerful chelating agents such as EDTA [99].

These results show that apo-MT, a strong *themodynamic sink* for Zn^2+^, neither sequesters much zinc in competitive reactions with the native Zn-proteome, nor pulls Zn^2+^ into cells via ZIP transporters to saturate its binding sites. Nevertheless, when cells were exposed to elevated extracellular Zn^2+^, increasing concentrations of MT were synthesized that accommodated extra Zn^2+^ entering the cells [61]. In this instance, the production of apo-MT served to protect cell viability. What does the existence of cellular apo-MT signify for the mechanism of zinc trafficking under conditions of normal zinc flux into cells?

It is evident that the presence of a substantial concentration of this powerful ligand for Zn^2+^ does not compete with and inhibit the normal trafficking pathway that conducts Zn^2+^ from the plasma membrane to apo-proteins that require Zn^2+^ for their function. A hypothesis that rationalizes this observation is based on Figure 2, and posits that MT acts as an intermediate in the trafficking of Zn^2+^ to Zn-proteins (Figure 4). As it delivers Zn^2+^ to apo-proteins, MT undergoes a cycle, moving between Zn-MT and apo- or unsaturated-MT. The concentration of Zn^2+^ bound to MT, Zn*_n_*-MT (*n* = 1–7), depends on the relative velocities of the reactions that shift Zn^2+^ into apo-protein (*V*_2_) and restore Zn^2+^ to apo-MT (*V*_1_). Differences in *V*_1_ and *V*_2_ in various cells would explain the observed variation in saturation of MT with Zn^2+^. Furthermore, *V*_1_ is expected to be dependent on the activity of ZIP and ZnT transporters that facilitate the transport of Zn^2+^ between the external medium and the cell interior. Thus, the relative saturation of the MT pool with zinc is likely to be an integrative outcome of the relative rates of the various zinc trafficking reactions shown in Figure 4 including zinc transport.

In order to test this hypothesis, the degree of Zn^2+^-saturation of MT was investigated as a function of the proliferative state of TE671 cells [94]. Normally, a steady-state pool of apo-MT exists in the cells, which double about every 24 h. Exposure to thymidine suppressed cell proliferation. In the process apo-MT was converted to Zn-MT. As the requirement for Zn^2+^ declined, *V*_2_ decreased. Assuming *V*_1_ remained approximately unchanged, Zn*_n_*-MT would become favored over apo-MT, as was observed. In a second test of this hypothesis, Ehrlich cells contain a steady state pool of Zn_7_-MT (*V*_1_ > *V*_2_) [87]. Determination of the rate of *V*_2_ under zinc-restricted conditions in which *V*_1_ = 0 showed that the magnitude of V_2_ is sufficient to supply most or all of the Zn^2+^ needed to support the rate of doubling by control Ehrlich cells [86].

## 8. Oxidized Metallothionein and the Trafficking of Zn^2+^

Mammalian MT contains 20 cysteinyl amino acids that supply sulfhydryl group ligands that bind Zn^2+^ and other metal ions [19]. Besides its participation in zinc trafficking, the sulfhydryl groups provide MT with the potential to serve as an important cellular anti-oxidant. Numerous reports demonstrate this role under a variety of conditions of oxidative stress [21,22,65,66,70]. Most of these studies showed that the presence or lack of MT correlated with a reduction in, or enhancement of, oxidant damage, respectively. Others found that Zn*_n_*-MT undergoes thiol oxidation in the presence of oxidants [21]. For MT to play a significant role in intercepting reactive oxygen species, it needs to be able to cycle between reduced and oxidized forms. Glutathione (GSH) readily reduces disulfide bonds in oxidized MT and, thereby, provides the means to maintain a pool of reduced MT [100].

A complete analysis of the importance of MT in protection against oxidant damage needs to compare its concentration and activity with the other sources of sulfhydryl groups. Glutathione constitutes a major pool of 1–10 mM thiol [101]. The proteome, which is a target of oxidant damage, contains a similar concentration of sulfhydryl groups [21,64]. To the extent that cysteinyl residues in the proteome become oxidized, its zinc buffering capacity may also be compromised, as discussed above. When H_2_O_2_ was the oxidant, Zn-MT was especially reactive among these classes of thiol groups [21]. In contrast, the other pools were preferentially reactive with NO [64].

Zinc-bound sulfhydryl groups that participate in redox reactions release Zn^2+^ as the protein is oxidized. Considering the interest in Zn*_n_*-MT as a donor of Zn^2+^ to apo-Zn-proteins, and the concern that its large affinity for Zn^2+^ might restrict such reactions, the hypothesis has gained popularity that the availability of zinc bound to MT for such reactions is dependent on the oxidation of zinc-metallothionein by the oxidized form of GSH, GSSG [38].

Zn_7_,S_20_-MT + 10GSSG **⥂** MT-(S-S)_10_ + 7Zn^2+^ + 20GS^−^ (16)

However, this pathway seems unlikely, both because the concentration of GSSG is ordinarily low in comparison with GSH and because the kinetics of Reaction (16) are very slow in contrast to the facile reaction of apo-MT with GSSG, and could not sustain timely mobilization of Zn^2+^ from MT [102]. Moreover, sole concern for this reaction as a means of delivering the metal to apo-Zn-proteins ignores the fact that Zn^2+^ liberated from MT-(S-S)_10_ in Reaction (16) immediately binds to the large concentration of high affinity, non-specific coordination sites in the proteome.

Zn^2+^ + Proteome_high affinity_**⥂** Proteome_high affinity_•Zn (17)

Once bound, Proteome_high_ _affinity_•Zn potentially becomes the source of Zn^2+^ for the putative constitution of Zn-proteins:

Proteome_high__affinity_•Zn + apo-Protein **⥂** Zn-Protein + Proteome_high affinity_ (18)

According to this scenario, MT and high affinity Proteomic sites collaborate in zinc trafficking.

## 9. Metallothionein Gene Expression

The facile induction of MT synthesis by Zn^2+^ and Cd^2+^ offers an example of trafficking that can be understood on the basis of a ligand substitution mechanism (Figure 1). Characterization of the determinants of metallothionein gene expression helped to usher in the modern era of eukaryotic molecular biology [103]. Of particular interest was the mechanism of induction of MT protein synthesis by metal ions including Zn^2+^. First, metal response elements (MREs) in the MT promoter region were discovered that conferred inducibility by zinc on the MT gene [104]. Then, a metal-response element-binding transcription factor, MTF-1, was identified [105]. It contains a MRE binding region comprised of six tandem Zn-fingers that utilize the common cys_2_his_2_ ligand set to bind Zn^2+^. Further, MTF-1’s DNA binding function was shown to be upregulated by Zn^2+^.

A simple model for MTF-1 activation was invoked, in which the protein gained the ability to bind to MRE DNA sequences upon conversion of MTF-1 into Zn*_n_*-MTF-1:

*n*Zn^2+^ + MTF-1 ⥂ Zn*_n_*-MTF-1 (19)

Experiments using lysates as a source of MTF-1 required µM additions of Zn^2+^ to stimulate MTF-1 binding to MREs [106]. This suggested that the conditional stability constants of the zinc-finger contingent of MTF-1 were modest and on the order of 10^6^ M^−1^ at pH 7.

The picture changed when direct measurements indicated that the conditional stability constants of component zinc-fingers of MTF-1 cluster about 10^11^ M^−1^ at pH 7 and it was recognized that the lysate used above was populated by strong coordination sites that competed with MTF-1 for Zn^2+^ [3,107]. As a robust Zn^2+^ binding ligand, MTF-1 could possibly react with pM-nM concentrations of free Zn^2+^ and might also be able to interact with Zn^2+^ associated with the proteome (*K*_Zn-proteome_ ≈ 10^10^) to undergo activation. Once the extracellular Zn^2+^ concentration returned to normal, the reversal of Reaction (19) seemed unlikely as the mechanism of deactivation, because the Zn^2+^ dissociation rate constants would be too small to support kinetically favorable loss of Zn^2+^ from the protein. Thus, sequestration of Zn^2+^ from Zn*_n_*-MTF-1 (*n* = 1–6) must involve ligand substitution, in which the product of Zn*_n_*-MTF-1 stimulated gene expression, apo-MT, or components of the proteome compete for Zn^2+^ to inactivate it (Reactions (20) and (21)):

Zn*_n_*-MTF-1 + apo-MT ⥂ MTF-1 + Zn*_n_*-MT  (*n* = 1–6) (20)

Zn*_n_*-MTF-1 + *n*Proteome_high affinity_ ⥂ MTF-1 + *n*Proteome_high affinity_•Zn (21)

Both apo-MT and Proteome_high affinity_ bind Zn^2+^ with sufficiently large stability constants and/or concentrations to compete for zinc bound to Zn*_n_*-MTF-1.

Supporting the involvement of apo-MT is the observation that the concentration of MT in the nucleus, the preferential location of zinc-activated MTF-1, increases following its induction by extracellular Zn^2+^ [108,109]. A possible complication for this reaction lies in the finding with another zinc-finger protein, Zn_3_-Sp1, that association with DNA markedly decreased its reactivity in model ligand substitution reactions [97]. If that is the case with Zn*_n_*-MTF-1, then the rate of dissociation of Zn*_n_*-MTF-1•MRE may act as the rate limiting step in the deactivation of this protein.

MTF-1 activation also occurs in cells exposed to cadmium ions. Originally, it was thought that Cd^2+^ like Zn^2+^ might activate the transcription factor directly through reaction with the apo-zinc finger domains of the protein. However, in vitro DNA binding assays showed that Cd^2+^ inhibited the MTF-1 interaction with metal response element DNA [110]. Moreover, according to structures of model peptides, Zn- and Cd-finger structures are subtly different such that the DNA recognition helix of the Cd-finger cannot make effective contact with cognate DNA [111]. Thus, it was proposed that Cd^2+^ reacts with Zn*_n_*-MT, causing the displacement of Zn^2+^ and making it available for reaction with MTF-1 [112]:

*n*Cd^2+^ + Zn*_n_*-MT ⥂ Cd*_n_*-MT + *n*Zn^2+^ (22)

Missing from this hypothesis was the recognition that the concentration of Zn*_n_*-MT may not be significant in some cells; yet induction of MT biosynthesis occurs upon their exposure to Cd^2+^. In these, and perhaps in most, cells, Zn^2+^ may be labilized for reaction with MTF-1 through metal ion exchange reactions between Cd^2+^ and Zn-proteins (Zn-proteome). Proteomic studies have shown that much of the native Zn-proteome can exchange with Cd^2+^ resulting in the liberation of Zn^2+^ from native zinc-binding sites and its subsequent adventitious association with high affinity, adventitious coordination sites in the proteome [53]:

Cd^2+^ + Zn-Proteome ⥂ Cd-Proteome + Zn^2+^ (23)

Zn^2+^ + Proteome ⥂ Proteome•Zn (24)

Proteome•Zn + MTF-1 ⥂ Zn-MTF-1 + Proteome(25)

In this mechanism, Proteome•Zn serves as the donor of Zn^2+^ to MTF-1 (Reaction (25)). Based on this example, it seems evident that viable models of signaling processes that utilize MTF-1 or other proteins to detect variable concentrations of Zn^2+^ will need to envision mechanisms that work within the strong Zn^2+^ binding environment provided by the proteome and apo- or unsaturated-MT.

## 10. Interaction of Metallothionein and the Proteome during Zinc Trafficking

**Figure 5:**
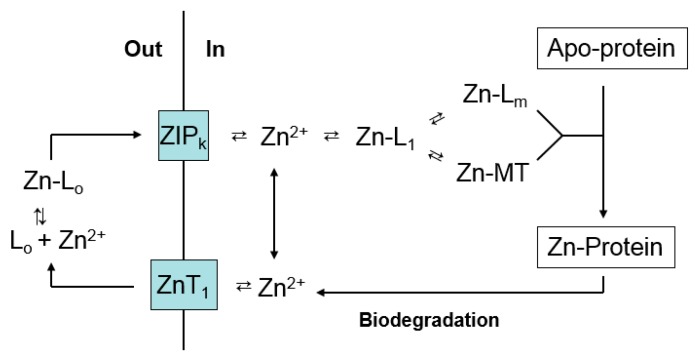
Zinc trafficking linking proteomic and MT binding sites for Zn^2+^.

A number of studies demonstrate that upon entering cells, Zn^2+^ faces a complex, potent ligand environment. This array of binding sites has been described as a buffer for *free* Zn^2+^ and a muffler to further reduce the availability of *mobile* Zn^2+^ [57]. Within this maze, pathways by which Zn^2+^ reaches apo-zinc proteins must also be present (Figure 2 and Figure 4). Although emphasis has been placed on metallothionein as a donor of Zn^2+^ to such structures, the survival of MT-null cells and organisms firmly indicates that other general routes of trafficking exist. With the additional recognition that both proteome and MT bind Zn^2+^ strongly, the involvement of proteomic-based Zn^2+^ trafficking must be considered. Nevertheless, the clear involvement of MT in the metabolism of zinc under a variety of circumstances, for example under conditions of cell proliferation or zinc deficiency as described above, points to the need to integrate both proteome and MT into a model of zinc trafficking. Figure 5 merges Figure 2 and Figure 4 to offer a hypothetical view of zinc trafficking that incorporates both proteome (L_1-m_) and MT as binding sites for Zn^2+^ as it traverses the cell from plasma membrane to apo-zinc protein.

The model considers MT as part of the proteomic manifold of ligands for Zn^2+^ and makes several assertions: (i) the pathway that utilizes L_1-m_ can operate without metallothionein; (ii) L_1-m_ and MT interact by ligand substitution during the handling of Zn^2+^; and (iii) Zn-MT has some special roles to play in the distribution and movement of intracellular Zn^2+^ that merit singling it out in the model. Examining each in turn, MT-null cells distribute Zn^2+^ satisfactorily; thus, proteomic pathways involving L_1-m_ exist to mediate the intracellular movements of Zn^2+^. How might they be revealed and studied?

A zinc sensor has been devised that employs plasmid expressed apo-carbonic anhydrase (apo-CA) as the target binding site for Zn^2+^ and a sensitive fluorescent probe that selectively forms a ternary complex with Zn-CA [59]. Using apo-CA instead as a model apo-zinc protein, its reaction with Zn^2+^ and the fluorophore, dansylamide (DNSA), has been investigated in the presence of supernatant from LLC-PK_1_ cells (Mahim and Petering, unpublished):

Apo-CA + Proteome_high__affinity_•Zn + DNSA ⥂ DNSA-Zn-CA + Proteome_high affinity_ (26)

Like apo-MT, apo-CA poorly competes with the native Zn-proteome for Zn^2+^ despite its favorable conditional stability constant of 10^11.4^ relative to at least some Zn-proteins [10,33,59]. Reaction (26) occurred under conditions in which the high affinity proteomic binding sites were in substantial excess of apo-CA, and virtually all of the added Zn^2+^ was initially bound to the proteome. Evidently, a kinetic pathway existed for tightly bound proteomic zinc to react with the target apo-protein.

Zn_7_-MT also donates Zn^2+^ to apo-CA and may be the preferable source when it is present in the cell [26,32]. Because proteome and MT both represent strong binding sites for Zn^2+^ that are kinetically reactive in ligand substitution reactions, the possibility must be considered that trafficking Zn^2+^ is distributed among these two pools. In a test of this idea, LLC-PK_1_ cells were incubated for 24 h with elevated Zn^2+^ (30 µM) to stimulate metallothionein synthesis without compromising proliferation (Mahim and Petering, unpublished). Then, the distribution of Zn^2+^ within cell supernatant was determined. As expected, MT-bound zinc was observed. In addition, the amount of Zn^2+^ in the proteome fraction increased and constituted 25% of the extra metal ion that was transported into the cells. At least in this circumstance, proteome as well as MT participates in the trafficking of zinc. Whether they represent independent pools or are coupled through metal exchange remains to be determined.

## 11. Emerging Technology to Address Zinc Trafficking

A metallothionein-proteome axis seems to be emerging as the basis for zinc trafficking. To achieve a more refined understanding of the roles of each, studies at the level of individual proteins will be needed. A promising analytical approach links laser ablation inductively coupled plasma mass spectrometry (LA-ICP-MS) with high-resolution separation of metalloproteins by polyacrylamide gel electrophoresis (PAGE) [11]. The methodology has foundered because of the lack of an adequate, native method of separation. One- or two-dimensional sodium dodecylsulfate PAGE (SDS-PAGE) methods provide excellent separation of proteins but do so at the cost of their denaturation. Recently, the formulation of native SDS-PAGE (NSDS-PAGE) demonstrated that high-resolution protein separation can be maintained together with retention of protein-bound metals [113]. This has raised hopes that LA-ICP-MS can be used as a highly sensitive, isotope specific monitor of protein-bound Zn^2+^.

At the same time, a means of probing Zn^2+^ bound adventitiously to the proteome has emerged from observations that the zinc fluorescent sensors, TSQ and Zinquin (ZQ), primarily detect Zn-proteins in cells through the formation of ternary adducts [114,115,116].

Zn-Protein + ZQ/TSQ ⥂ TSQ/ZQ-Zn-Protein(27)

Moreover, when extra Zn^2+^ was added to the proteome, both TSQ and ZQ formed adducts with non-specifically bound zinc [51,117]. Using this information, an affinity bead has been fashioned that employs ZQ as the ligand [118]. With this tool, proteomic samples highly enriched in Zn-proteins have been isolated. Extending this method to the isolation of protein•Zn complexes should provide new options for studying zinc trafficking and the interaction of members of the proteome with metallothionein.
